# Association between Socioeconomic Status and Vision Screening Outcomes among Preschool Children in Klang Valley, Malaysia: A Cross-Sectional Study

**DOI:** 10.21315/mjms2022.29.2.10

**Published:** 2022-04-21

**Authors:** Humairah KAMARUDDIN, Naufal NORDIN, Nurlin Erlina ABDUL MANAP, Sumithira NARAYANASAMY, Sharanjeet SHARANJEET-KAUR, Mohd Izzuddin HAIROL

**Affiliations:** 1 Centre for Community Health Studies (ReaCH), Faculty of Health Sciences, Universiti Kebangsaan Malaysia, Kuala Lumpur, Malaysia; 2 Centre for Rehabilitation & Special Needs Studies (iCaReRehab), Faculty of Health Sciences, Universiti Kebangsaan Malaysia, Kuala Lumpur, Malaysia

**Keywords:** vision screening, socioeconomic status, preschool children, visual acuity, stereopsis

## Abstract

**Background:**

Vision screening programmes’ outcomes are routinely used to report the prevalence of vision anomalies in children. However, the association between vision screening outcomes and the children’s socioeconomic status remains underexplored. This cross-sectional study determined the association between socioeconomic and birth status with vision screening outcomes in a sample of children in Klang Valley.

**Methods:**

Total 411 children (mean age: 5.49 ± 0.47 years old) attending preschools were selected via stratified cluster sampling. Habitual distance visual acuity, near visual acuity, and stereoacuity were measured. The fail criteria were distance visual acuity ≥ 0.3 logarithm of the Minimum Angle of Resolution (logMAR), near visual acuity ≥ 0.4 logMAR or stereoacuity ≥ 300 arcsec. Socioeconomic and birth history data were obtained using parent-report questionnaires. The association between socioeconomic factors and screening outcomes were determined with binary logistic regression.

**Results:**

Sixty-two children (15.1%) failed the screening, with a significantly higher failure rate for Bumiputera children (16.34%) compared to non-Bumiputera children (4.08%) (*χ*^2^_(1, 410)_ = 5.21; *P* = 0.024). After adjusting for confounders, Bumiputera children were four times more likely to fail vision screening (OR: 4.54; 95% confidence intervals [CI]: 1.07, 17.76; *P* = 0.044). Other socioeconomic factors were not significant predictors for failing vision screening.

**Conclusion:**

Preschool children’s ethnicity is associated with vision screening outcomes. Bumiputera children are more likely to fail vision screening than their non-Bumiputera peers.

## Introduction

Early comprehensive eye and vision examination should be considered for children aged between 3 years old and 5 years old (1), as effective treatment and intervention for any visual anomalies in young children may improve the children’s visual-motor function (2) and educational performance (3). It has been reported that 45%–68% of parents never take their child to a full eye examination (4–7). In Malaysia, the coverage of vision screening is limited for preschool children, where priority is given to those attending government and government-aided schools only (8). As up to 7% of children attend preschools while having blurred distance vision (9), effective and timely vision screening programmes may aid the detection and treatment of visual anomalies.

In adult populations, a significant association has been reported between low socioeconomic status and visual impairment among gender and ethnic groups (10). Individuals from low household income and poor educational attainment were at high risk for visual impairment in both urban and rural regions (11). Specifically for children, those from the most disadvantaged backgrounds were reported more likely to suffer from undetected vision problems (12–14) and reduced visual-motor integration skills (15). However, children from socioeconomically secure families also reported unnoticed vision problems (9, 16). Therefore, the assumption that the inequity of access to health care results in vision problems is inconclusive, as vision defects may arise regardless of children’s socioeconomic backgrounds.

While several studies have examined the prevalence of vision problems among preschool children in Malaysia (17–19), research on the association between vision problems and the socioeconomic status of these children remains to be explored. In addition, the vision parameter measured was mainly habitual visual acuity and did not consistently include other important measures such as near acuity and stereoacuity. The primary objective of this study was to identify the socioeconomic factors associated with the outcomes of vision screening in preschool children in Klang Valley, Malaysia. The other objective of this study was to report the outcomes of vision screening in preschool children that consisted of the measurements of distance visual acuity, near visual acuity and stereoacuity. This study may provide an overview of the demographic and socioeconomic influences on preschool children’s visual outcomes in urbanised areas. This information could be valuable to quantify the need to implement a vision screening program targeted towards specific groups within the population and identify priority research areas.

## Methods

This cross-sectional study was conducted from September 2019 to February 2020 and involved 5-years-old and 6-years-old children with no reported physical, pathological or cognitive disabilities and who attended selected public or private preschools located in Klang Valley. The sample size was calculated based on 136,032 live births recorded in 2014 for Selangor and the Federal Territory of Kuala Lumpur (20). This population was anticipated to enrol in preschool during the data collection period. Krejcie and Morgan (21)’s formula, with the desired confidence level of 95% and precision of ±5, was used to calculate a minimum sample size of 380. Participants were recruited using stratified cluster sampling. The preschools were stratified by type, i.e. public and private preschools. The list of public and private preschools was accessible through the Community Development Department (KEMAS) (22) and Ministry of Education Malaysia (23), respectively. For each type of preschool, they were selected using simple random sampling, and all students from the selected preschools were included as participants.

### Study Parameters

A structured and self-administered questionnaire was distributed to the parents or legal guardians to collect data on the child’s demographic and socioeconomic characteristics. The items assessed were defined by two categories for each variable of interest, which covered: i) the children’s demographics: gender (male or female), ethnicity (Bumiputera or non-Bumiputera), birth history (normal: full-term born or born without complication, or abnormal: preterm born or born with complication), preschool enrolment age (before 5 years old or ages 5 years old or older), type of preschool (public or private) and ii) children’s family socioeconomic status: parental educational level (secondary education and below, or above secondary education), parental employment status (employed or unemployed), monthly household income (less than RM3,000 per month or RM3,000 and above per month), and sibship size (3 or less, or more than 3) and living area (urban or suburban). The income of RM3,000 was taken as the cut-off point as it was the median household income for the bottom 40% of household earners in Malaysia (24). Based on the provided address, classification of urban and suburban areas was based on whether the respondents were living under jurisdiction of the City Council (*Majlis Bandaraya*) or the Municipal Council (*Majlis Perbandaran*), respectively (25).

The vision screening programme consisted of habitual distance visual acuity measurements, near visual acuity and stereoacuity testing. Habitual distance and near visual acuity were assessed for each eye using the LEA Symbols^®^ Pediatric Test Book and LEA Symbols^®^ Near Vision Card (Good-Lite Co, Elgin, IL), respectively. The total number of symbols that correctly names or matches was recorded as the logarithm of the Minimum Angle of Resolution (logMAR) notation. A combination of distance and near visual acuity test was reported to be more accurate for detecting significant refractive errors rather than either of the two tests alone (26). Stereopsis was assessed using the Frisby Stereotest (Frisby Stereotests, Fulwood, United Kingdom). As poor stereopsis is often linked to strabismus and amblyopia (27–28), the stereoacuity test was included in the screening. All procedures were performed in the preschools’ classrooms under sufficient room lighting by a postgraduate researcher with a Bachelor’s degree in Optometry and assisted by five undergraduate Optometry students. The vision screening was monitored and validated by three supervisory team members, all with more than 10 years of experience in the paediatric optometry field.

### Classification of Vision Screening Outcomes

In this study, the vision screening outcomes were categorised as pass or fail. A child was classified to have failed the vision screening if they failed at least one of the three vision screening elements. The fail criteria for distance visual acuity were 0.3 logMAR and worse in either eye or both eyes (29–30). The fail criteria for near visual acuity were 0.4 logMAR or worse in either eye or both eyes (31–32). The fail criteria for stereopsis were stereoacuity of 300 arcsec or worse with the Frisby stereotest (33).

### Statistical Analysis

All data were analysed using the IBM SPSS version 25.0 (IBM Corp, Armonk, New York, USA). Multiple imputations were conducted to handle any missing data, preserve the sample size and produce unbiased estimates (34–35). Twenty imputed datasets were generated under a multivariate normal model to reduce sampling variability from the imputation process (36–37). Descriptive analysis was used to assess the distribution of preschool children’s demographic and socioeconomic characteristics and vision screening outcomes. Comparative analyses were conducted using the Pearson’s Chi-square test or Fisher’s exact test, as appropriate. The predictor variables that produced a *P*-value of 0.20 or below were included in binary logistic regression models. Binary logistic regression was performed to assess the socioeconomic risk factors on the likelihood that participants would fail the vision screening. The first regression model was unadjusted, where the confounding variables were analysed separately. The second model was adjusted by the confounding variables. The significance level was determined at *P* < 0.05 with 95% confidence intervals (CI).

## Results

The mean age of the 411 participants was 5.94 ± 0.47 years old (range: 5.08 to 6.83 years old) and males comprised 52.6% of the participants. The majority of participants were ethnically Malay (*n* = 344 [83.9%]). The number of indigenous children was combined with Malay children and categorised as Bumiputera (*n* = 361 [88.0%]), while children from other ethnicities (Chinese, Indians and others) were categorised as non-Bumiputera (*n* = 49 [12.0%]). This was done for two reasons: i) it is the legal ethnic classification as outlined by the country’s constitution and ii) the number of participants of each non-Bumiputera ethnicity was relatively small (between 3.2% and 6.3%). Table 1 summarises the distribution of sociodemographic characteristics of the study participants.

**Figure 1:**
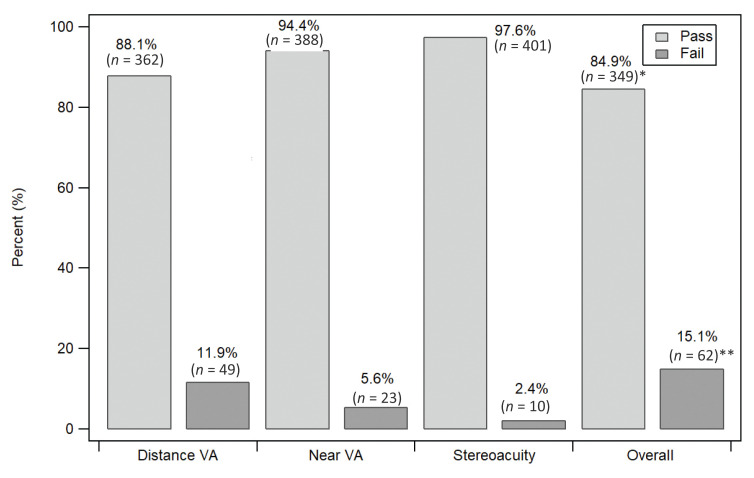
Vision screening assessment

The results of vision screening are shown in Figure 1. Eighty-eight percent of the children passed the distant visual acuity test, 94.4% passed the near visual acuity test and 97.6% passed the stereoacuity test. Overall, 84.9% of children passed all three vision assessments. Table 2 summarises the distribution of vision screening outcomes based on the participants’ demographic and socioeconomic characteristics. A significantly higher proportion of Bumiputera children failed the vision screening (16.34%) compared to non-Bumiputera children (4.08%) [*χ*^2^ (1, 410) = 5.21; *P* = 0.02)]. Four predictor variables had a *P*-value of 0.20 or below (ethnicity, birth history, paternal employment status and maternal employment status). Therefore, these variables were included in the binary logistic regression analyses.

Table 3 shows the odds ratios of the logistic regression models. In the unadjusted model, the four confounding variables were analysed separately. In the adjusted model, all the factors were included together in the analysis. With the adjusted model, it was found that Bumiputera children were four times more likely to fail vision screening [OR: 4.54; 95% CI: 1.07, 17.76; *P* = 0.044]. Meanwhile, the other demographic and socioeconomic factors were not significant predictors for failing vision screening.

## Discussion

Overall, 15.1% of the children who participated in this study failed at least one of the three assessments conducted under vision screening. Our findings are favourable with the outcomes of earlier studies in Malaysia, where 12.5% of preschool children in Segamat District had visual acuity worse or equal than 0.25 logMAR (18) and 9% of preschool children in Manjung, Perak failed vision screening (17). However, only 5% of preschool children in urban Kuching, East Malaysia, had distant visual acuity worse than or equal to 6/12 in one or both eyes measured with the Sheridan Gardiner chart (38). The lower incidence rate of reduced vision in young children is possibly related to using a letter chart, instead of symbols, for acuity screening. Indeed, the Lea Symbols chart offers a better visual impairment detection rate for preschool vision screening than the Sheridan Gardiner chart (30, 39). The results reported in this study compared well with those of other countries, where the failure rate was 11.9% for children aged 4 years old–5 years old in Scotland (12) and up to 19% failure rate for children in economically disadvantaged areas of New York City (40). However, when measured with the Sheridan Gardiner chart, only 2.7%–3.8% of Hong Kong children aged 2 years old–6 years old presented with visual acuity worse than or equal to 6/12 (41).

The current study found that Bumiputera children had a significantly higher risk of failing vision screening, indicating the presence of some form of uncorrected refractive error. In an earlier study, it was found that non-Bumiputera children had a significantly higher odds ratio (up to 7.98) for having a refractive error (42). Although most refractive error variation within a population is thought to be due to genetics, several studies agreed that environmental factors might also be crucial in determining individual risks of refractive error (43–45). Evidence of this interaction may be seen in a previous study on the prevalence of refractive error among children between ethnicities in Singapore and Malaysia. Regardless of their ethnicity, Singaporean children had a higher prevalence of refractive errors than their Malaysian peers (46). This finding suggests that environmental factors may contribute to the higher rates of refractive error rather than genetics alone.

Prematurity has been associated with increased vision problems in preterm populations (47–48). Thus, these children may have a higher odds of failing vision screening. Preterm infants possess a higher risk of amblyopia, strabismus and uncorrected refractive error compared to full-term infants when they reach six years old of age (49). However, the current study did not find a significant association between these measures. It is worth noting that Malaysian Clinical Practice Guidelines on Retinopathy of Prematurity asserts that premature infants should be screened for their ocular conditions after 4 weeks–6 weeks of birth and periodically monitor its progress till they reach preschool years, if applicable (50). This indicates a reality wherein our systems and healthcare facilities are available for early detection, intervention and ongoing support for high-risk populations, specifically in preterm infant groups.

The current study did not find significant associations between parental employment status and children’s vision screening outcomes. Indeed, a previous study did not find a statistically significant association between parental employment status and their commitment to seeking a comprehensive eye examination after their child had failed visual acuity screening (51). Pieters and Rawlings (52) reported that the unemployment of a father was a disadvantage for their child’s health, but the unemployment of a mother was beneficial to their child’s health status. It was suggested that having unemployed fathers would, on average, reduce family income, creating barriers to obtaining health care for their children. Meanwhile, employed mothers would be required to reorient their time to meet their dual demands of working and childcare, which could significantly impact child health outcomes (53–54). Our present data could not address these hypotheses as the current study found no significant associations between parental employment status and children’s vision screening outcomes.

Although the present study includes a population-based design and standardised vision assessments, several limitations were subjected to this study. Firstly, the study derived the socioeconomic status of participants based on parent-reported questionnaires, which might not accurately depict the actual socioeconomic status of the sample. Parents or legal guardians might misreport their response to several items that were seen as their personal worth, such as educational level, employment status and income (55–56). Classification of a child’s ethnicity was determined based on the questionnaires filled in by their parents, which could be questionable in the case of mixed-parentage. However, they were provided with the option of ‘Others’ if their children did not classify as Malay/indigenous, Chinese or Indian ethnicity. In addition, the current study’s questionnaire grouped those born preterm or with complications as having an abnormal birth history, where these conditions may affect children’s development differently (57–58). However, with the relatively low number of participants who indicated that the birth was preterm or with complications (n = 24 [8.2%]), the analysis outcome was unlikely to change had the preterm and complicated birth data were categorised separately. Secondly, a large percentage of the participants were Bumiputera children, which might have skewed the study’s findings and affected the power of the study, widening the 95% confidence interval for the odds ratio (59). The samples’ ethnicity also did not reflect the ethnicity distribution of the Malaysian population. Thus, generalisation of the results is not possible. In a future study, the ethnicity proportion of the sample should be made to reflect the ethnicity proportion of the population. Furthermore, the study did not measure the refractive error of the participants and probably underestimated the vision screening outcomes. This could be a disadvantage for children who may have had latent hyperopia, astigmatism and low myopia since the cut-off values used in the visual acuity screening do not reliably detect them (60–61). Hence, performing cycloplegic refraction should be considered to improve the detection rate of uncorrected refractive errors among preschool children in a future study.

## Conclusion

Preschool children’s ethnicity is associated with vision screening outcomes, where Bumiputera children are more likely to fail vision screening tests than their non-Bumiputera peers. However, other demographic and socioeconomic factors are not significant predictors for the outcomes of vision screening.

## Figures and Tables

**Table 1:** The distribution of the study participants’ socioeconomic characteristics and birth history status

Variables	*N* (%)
Gender	
Male	216 (52.6)
Female	195 (47.4)
Ethnicity	
Bumiputera	361 (88.0)
Non-Bumiputera	49 (12.0)
Birth history	
Normal	267 (91.8)
Abnormal	24 (8.2)
Preschool type	
Public	232 (56.4)
Private	179 (43.6)
Preschool enrolment age	
3 years old–4 years old	85 (21.1)
5 years old–6 years old	318 (78.9)
Maternal education level	
Secondary education and below	215 (53.6)
Above secondary education	186 (46.4)
Paternal education level	
Secondary education and below	240 (60.9)
Above secondary education	154 (39.1)
Maternal employment status	
Employed	266 (66.5)
Unemployed	134 (33.5)
Paternal employment status	
Employed	390 (98.7)
Unemployed	5 (1.3)
Household Income	
< RM3,000	267 (65.4)
≥ RM3,000	141 (34.6)
Living area	
Urban	343 (83.9)
Suburban	66 (16.1)
Sibship size	
1–3	304 (74.0)
4 and above	107 (26.0)

**Table 2:** Socioeconomic characteristics and birth history status of participants based on vision screening outcomes

Variables	Pass *n* (%)	Fail *n* (%)	Chi-square statistic *(df)*	*P-*value
Gender				
Male	183 (84.7)	33 (15.3)	0.01 (1)	0.909
Female	166 (85.1)	29 (14.9)		
Ethnicity				
Bumiputera	302 (83.7)	59 (16.3)	5.12 (1)	0.024*
Non-Bumiputera	47 (95.9)	2 (4.1)		
Birth history				
Normal	236 (88.4)	31 (11.6)	-	0.196†*
Abnormal	19 (79.2)	5 (20.8)		
Preschool type				
KEMAS (public)	201 (86.6)	31 (13.4)	1.24 (1)	0.266
Private	148 (82.7)	31 (17.3)		
Preschool enrolment age				
3 years old–4 years old	73 (85.9)	12 (14.1)	0.05 (1)	0.822
5 years old–6 years old	270 (84.9)	48 (15.1)		
Maternal education level				
Secondary education and below	184 (85.6)	31 (14.4)	0.11 (1)	0.743
Above secondary education	157 (84.4)	29 (15.6)		
Paternal education level				
Secondary education and below	205 (85.4)	35 (14.6)	0.20 (1)	0.656
Above secondary education	129 (83.8)	25 (16.2)		
Maternal employment status				
Employed	221 (83.1)	45 (16.9)	2.29 (1)	0.130*
Unemployed	119 (88.8)	15 (11.2)		
Paternal employment status				
Employed	331 (84.9)	59 (15.1)	-	0.172†*
Unemployed	3 (60.0)	2 (40.0)		
Household income				
< RM3,000	231 (86.5)	36 (13.5)	1.31 (1)	0.253
≥ RM3,000	116 (82.3)	25 (17.7)		
Living area				
Urban	292 (85.1)	51 (14.9)	0.14 (1)	0.709
Suburban	11 (50.0)	11 (50.0)		
Sibship size				
1–3	255 (83.9)	49 (16.1)	0.97 (1)	0.324
4 and above	94 (87.9)	13 (12.2)		

Notes:

† Fisher’s exact test;

* Significant at *P* < 0.2 and parameters are included in binary logistic regression analysis

**Table 3:** Factors associated with failed vision screening in a sample of Malaysian preschool children

	Unadjusted model			Adjusted model		

	OR	95% CI	*P*-value	OR	95% CI	*P*-value
Ethnicity						
Non-Bumiputera	1.00			1.00		
Bumiputera	4.57	1.08, 19.36	0.039*	4.54	1.07, 17.76	0.044*
Birth history						
Normal	1.00			1.00		
Abnormal	2.00	0.74, 5.40	0.167	2.00	0.65, 4.78	0.263
Maternal employment status						
Unemployed	1.00			1.00		
Employed	1.58	0.84, 2.96	0.153	1.67	0.82, 2.95	0.179
Paternal employment status						
Employed	1.00			1.00		
Unemployed	1.86	0.35, 9.80	0.464	1.84	0.31, 13.04	0.462

Note:

* OR is significant at *P* < 0.05
